# Strategies for Molecular Imprinting and the Evolution of MIP Nanoparticles as Plastic Antibodies—Synthesis and Applications

**DOI:** 10.3390/ijms20246304

**Published:** 2019-12-13

**Authors:** Doaa Refaat, Mohamed G. Aggour, Ahmed A. Farghali, Rashmi Mahajan, Jesper G. Wiklander, Ian A. Nicholls, Sergey A. Piletsky

**Affiliations:** 1 Department of Pathology, Animal Health Research Institute (AHRI), Agricultural Research Center (ARC), Giza 12618, Egypt; vet_dr_doaareffat@yahoo.com; 2 Department of Materials Science and Nanotechnology, Faculty of Postgraduate Studies for Advanced Sciences (PSAS), Beni-Suef University, Beni-Suef 62511, Egypt; ahmedfarghali74@yahoo.com; 3 Department of Biotechnology, Animal Health Research Institute (AHRI), Agricultural Research Center (ARC), Giza 12618, Egypt; galalpasha@yahoo.com; 4 Bioorganic & Biophysical Chemistry Laboratory, Linnaeus University Centre for Biomaterials Chemistry, Department of Chemistry & Biomedical Sciences, Linnaeus University, SE-39182 Kalmar, Sweden; rashmi.mahajan@lnu.se (R.M.); jesper.wiklander@lnu.se (J.G.W.); 5 Chemistry Department, College of Science and Engineering, University of Leicester, Leicester LE1 7RH, UK

**Keywords:** assay, molecular imprinting, nanoMIP, protein imprinting, sensor, therapeutic agent

## Abstract

Materials that can mimic the molecular recognition-based functions found in biology are a significant goal for science and technology. Molecular imprinting is a technology that addresses this challenge by providing polymeric materials with antibody-like recognition characteristics. Recently, significant progress has been achieved in solving many of the practical problems traditionally associated with molecularly imprinted polymers (MIPs), such as difficulties with imprinting of proteins, poor compatibility with aqueous environments, template leakage, and the presence of heterogeneous populations of binding sites in the polymers that contribute to high levels of non-specific binding. This success is closely related to the technology-driven shift in MIP research from traditional bulk polymer formats into the nanomaterial domain. The aim of this article is to throw light on recent developments in this field and to present a critical discussion of the current state of molecular imprinting and its potential in real world applications.

## 1. Introduction

Molecular recognition, the ability of systems to selectively recognize and bind complementary molecules present in complex mixtures, is the fundamental basis for all chemical and biological processes. Binding occurs via various forms of non-covalent interactions, such as hydrogen bonds, electrostatic interactions, hydrophobic interactions, and weak metal coordination [1]. Molecular imprinting is a strategy that entails the use of these types of interactions for the recognition of predetermined ligands by synthetic polymers, thus mimicking the recognition events observed in biomolecular recognition processes [2]. Molecular imprinting has become established as a mature technology with, currently, over 15,000 publications describing MIP synthesis, characterization, and use in a wide range of application areas [3]. The ligand-selectivities that can be observed in MIP-systems, together with their robust chemical nature, which is in particular due to their high degree of cross-linking and provides them with substantially more stability than biomolecular recognition species, e.g., antibodies, have driven research in this field [4,5,6].

**Figure 1:**
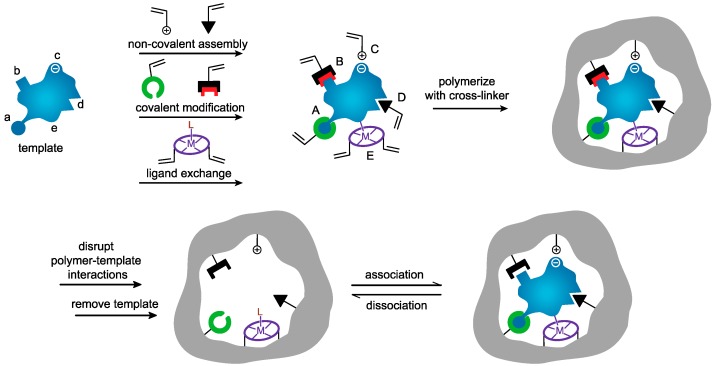
Schematic representation of the molecular imprinting process, reproduced from [16] with permission.

The molecular imprinting concept has a long history which traces back to the 1930s when the Soviet chemist Polyakov reported unusual adsorption properties of silica particles prepared in the presence of soluble additives [7]. The modern-day approaches to imprinting began in Europe in the 1970s and 1980s with Günter Wulff in Germany and Klaus Mosbach in Sweden [8,9]. Synthetic polymer-based imprinting strategies fall into three general classes; covalent, non-covalent, and semi-covalent imprinting protocols as defined by the nature of the interaction between the template and functional monomer(s) (T/M) [10] (Figure 1). The covalent approach yields a remarkably well defined and homogenous distribution of binding sites [11], while the non-covalent counterpart yields heterogenous binding sites [12,13,14]. The semi-covalent strategy is a hybrid of the former two, where the T/M binding and analyte rebinding occur via covalent and non-covalent chemistries, respectively [15].

The literature reports the synthesis of MIPs in formats suitable for different applications ranging from monoliths and membranes to films and beads [16]; however, a number of shortcomings have hindered their implementation in real-world applications. These drawbacks include recognition site heterogeneity, template leakage, mass transfer limitations, and solubilities. A step-change in the field has resulted from a shift of focus in MIP research from bulk polymers to nanomaterials, which has provided a strategy to address these issues [2]. A number of factors underlie the success obtained using MIP nanoparticles (nanoMIPs) to resolve the problems associated with bulk MIPs; notably, they possess larger surface/mass ratio, have more easily accessible recognition sites and, importantly, they have lower heterogeneities and better solubilities; factors which have been instrumental in their successful use in a diverse range of applications such as diagnostics, imaging and drug delivery [2,17]. This current review highlights the challenges faced when using bulk imprinting and the recent achievements in the development of nanoscale molecularly imprinted plastic antibodies along with their potential for use in real-world applications. 

## 2. Imprinting Challenges

MIPs have tremendous commercial potential; however, there is very little evidence of their successful application in solving real world problems. There are two main reasons behind this. The first one is associated with the dominance of antibodies in diagnostic and therapeutic applications. For MIPs, aptamers and other biomimetic materials it is very difficult to compete with well-established technologies that already have attracted multibillion-dollar investments [18,19,20]. The second reason is related to the technological challenges faced by traditional (bulk) molecular imprinting, particularly:

(i) Difficulty with imprinting of biological macromolecules, which are not soluble in organic solvents that are traditionally used in molecular imprinting. All bulk polymers, especially polymers imprinted with large templates such as proteins, also suffer from slow mass transfer kinetics. Protein recognition is the most important area of bioanalysis and drug development and for these reasons traditional MIPs are not considered as a viable alternative to antibodies.
(ii) Template leakage (bleeding) which affects analytical applications of MIP particles. It is not feasible to use MIP as a biorecognition material in assays and sensors if there is a risk that leaked template can compromise clinical or forensic analysis.
(iii) Heterogeneity of binding sites. Bulk MIPs always have large numbers of non-specific sites which contribute to the “polyclonal” nature of their binding profiles [12,21,22]. High levels of non-specific binding limit the utility of MIPs in diagnostic, pharmaceutical, and separation applications, except in a limited number of special cases where there is no alternative.

Herein, the significant recent attempts that have been made to address these challenges by producing MIPs in ‘nano’ formats and to improve their performance are presented.

### 2.1. Imprinting of Proteins

The molecular imprinting of proteins is necessary for the use of imprinted materials in many applications, such as diagnostics, drug delivery, environmental analysis, and proteomics [23]. Protein imprinting is a challenge due to the large size of protein molecules, their complexity, flexibility, and poor solubility in organic solvents [24]. Bulk imprinting, at first glance, appears the simplest approach for protein imprinting, with the objective of obtaining macroporous polymer networks which can entrap and release entire protein molecules. However, in the case of whole protein imprinting, conformational variation will lead to a multitude of different binding sites in an imprinted polymer, with different affinities and specificities. Accordingly, the product of protein imprinting will be polyclonal in character, due to the broad range of binding sites obtained [21]. Proteins are water-soluble substances representing a challenge for imprinting in organic solvents typical for the bulk imprinting process [21]. Furthermore, protein structure is sensitive to the non-physiological environment of radical polymerization such as the presence of organic solvent and functional monomers [25] and changes in temperature or pH [26]. Thus, polymerization in an aqueous environment is the preferred option. However, many popular monomers used in molecular imprinting are insoluble in water [24] and water may also compete with the template-monomers interactions reducing MIP affinity [27]. On the other hand, water provides an opportunity to explore hydrophobic interactions for template recognition [28,29]. The non-specific hydrophobic interactions can be reduced by using hydrophilic monomers and cross-linkers [30]. Proteins can be wasted by being trapped inside bulk polymers, which is particularly bad in case of expensive targets. Furthermore, both the surface chemistry and the pore sizes of the polymer can be affected by the extremely harsh conditions such as high temperature and strong acids necessary for at least partial removal of protein template entrapped in the polymer matrix, which can in turn impact adversely on selectivity and adsorption. To circumvent these challenges several other approaches have been developed to address these issues in bulk polymers, including; surface imprinting [31,32], epitope-mediated surface imprinting [33], and micro-contact imprinting [34].

**Figure 2:**
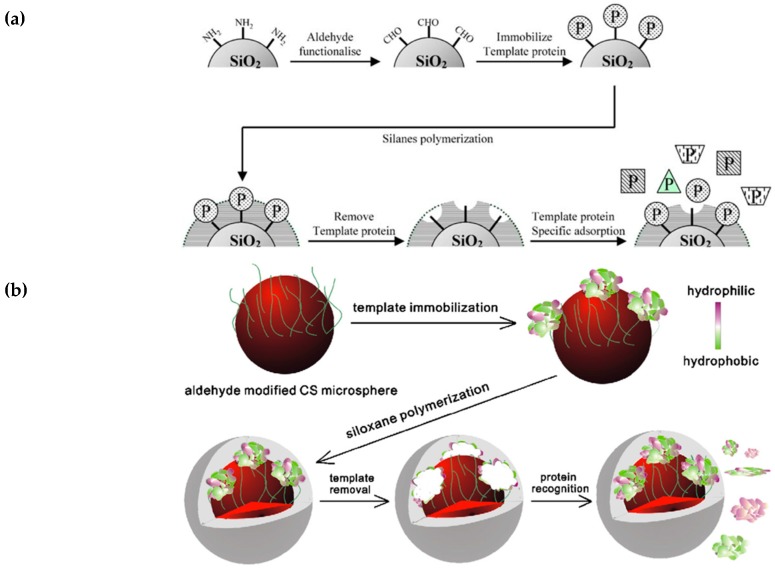

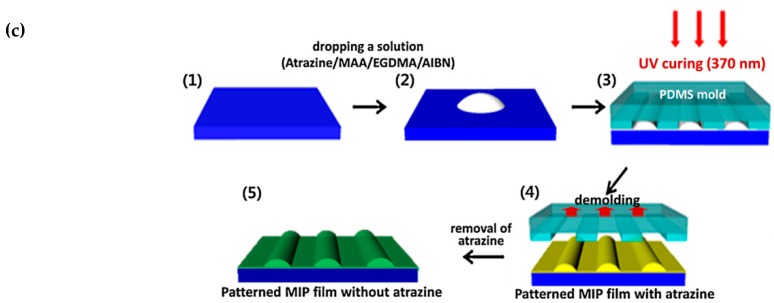
Schematic representations of protocols used in surface imprinting; (**a**) imprinting immobilized template on silica surfaces, reproduced from [35] with permission; (**b**) imprinting by surface grafting, reproduced from [36] with permission; and (**c**) soft lithography and UV initiated polymerization, adapted from [37] with permission.

**Figure 3:**
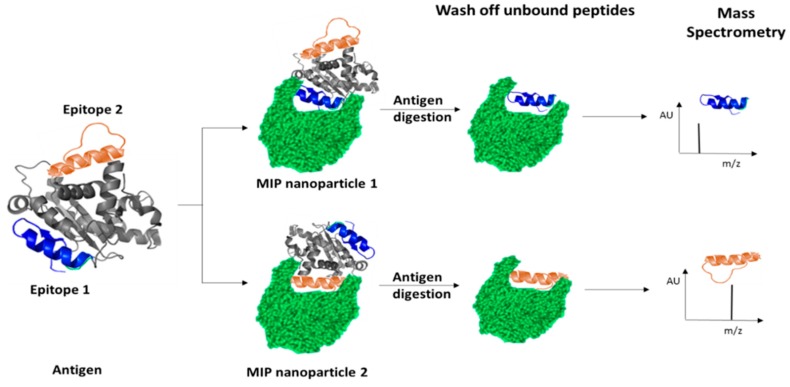
Epitope mapping: imprinting of the target protein (antigen), digestion of the protein to produce a peptide–polymer complex; isolation the peptide–polymer complex; and sequencing the attached peptide(s).

In surface imprinting (Figure 2), a certain degree of protein stabilization is achieved by using oriented immobilization of template [35,36,37,38]. Typically, template is immobilized onto the surface of a sacrificial material such as SiO_2_, which is immersed in the monomer mixture during polymerization. Following polymerization SiO_2_ is dissolved, leaving behind binding sites occupied by template. In the final step template is extracted from the polymer by extensive washing or hydrolysis [39,40]. In addition to stabilization of protein structure, template immobilization expands the range of solvents available for imprinting, thus allowing for substances insoluble in the polymerization mixture to be imprinted [41]. This also helps with achieving better control over conformation of created imprints through the control of template orientation [41], prevents protein aggregation [12] and facilitates the mass transfer kinetics [42,43]. Even this approach has associated challenges, including: poor control over the thickness of the polymer film, difficulty with extraction of the template from a dense polymer layer, potential leaking of the entrapped template from the polymer, and long time required to prepare MIPs [44]. Surface imprinting can be easily combined with different nanomaterial strategies where nanomaterials act as sacrificial molds, offering more precise control over the morphology of the imprinted polymer. This involves MIP synthesis inside a sacrificial porous nanomaterial which is then removed, leaving the nanostructured polymeric material, in the form of nanorods [45,46], nanofilaments [47] or ordered cavities [48]. The nano-structuring of the material provides significant enhancement of the MIP surface area and, consequently, improves sensitivity, detectability, and response time when used in a sensor format. NanoMIPs can also be synthesized by confining the polymerization reaction to the surface of nanoparticles made from silica [49], quantum dots [50], iron oxide [51,52], and alumina membranes [53,54]. However, the translation of surface imprinting from bulk polymers to nanoparticles through the grafting of MIP shells onto nanoparticle cores is much more difficult to control. Aggregation of nanoparticles, poor penetration of UV light through the dense suspension, and complicated purification procedures make this approach unsuitable for large-scale industrial applications. One option to avoid imprinting of costly or difficult to handle proteins is to instead imprint epitopes. In epitope-mediated imprinting, the whole protein template is replaced by a peptide fragment (typically 6–12 amino acids) characteristic of this protein [12,55]. In a model study, imprinting of cytochrome, alcohol dehydrogenase, and bovine serum albumin was achieved using C-terminal nonapeptides [33,56]. The epitope approach is superior to the other general techniques since it provides relatively easy template removal, generates uniform binding sites and reduces costs of synthesis, especially in the case of expensive protein templates [12,57]. The polypeptide templates are far less sensitive to the surrounding environments as they do not have secondary and tertiary structures [58]. The disadvantage of this approach is actually closely related to the major problem associated with production of antibodies, which lies in the complex procedure applied to finding appropriate (linear) epitopes. Prediction of epitope structure requires detailed knowledge of protein conformations [59]. Structural analysis by crystallography does not necessarily provide structures accurately representing native protein structure in solution as may be observed using NMR. A protocol for identification of protein epitopes suitable for molecular imprinting has recently been reported that can facilitate this process [60], as summarized in Figure 3.

**Figure 4:**
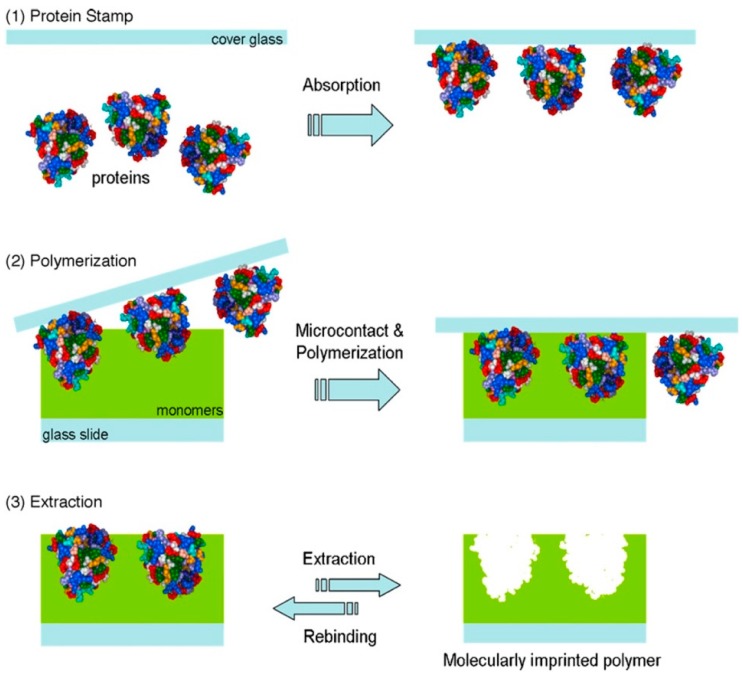
Schematic representation of micro-contact imprinting, reproduced from [61] with permission.

Micro-contact imprinting is considered as one of the promising techniques for protein imprinting [61] that can address its common associated problems such as solubility, conformational stability, and aggregation during the polymerization process [62]. This approach allows rapid fabrication of MIPs using small amounts of template, and monomer solution with a possibility to polymerize dozens of samples at the same time applying the same polymerization batch [62,63,64,65]. In a typical procedure, the template is firstly immobilized or adsorbed on a support (glass) to create the protein stamp. The stamp is subsequently set in contact with monomers followed by a polymerization step. The cover glass is then removed yielding an imprinted thin film (Figure 4).

### 2.2. Incomplete Template Removal and Template Leakage

**Figure 5:**
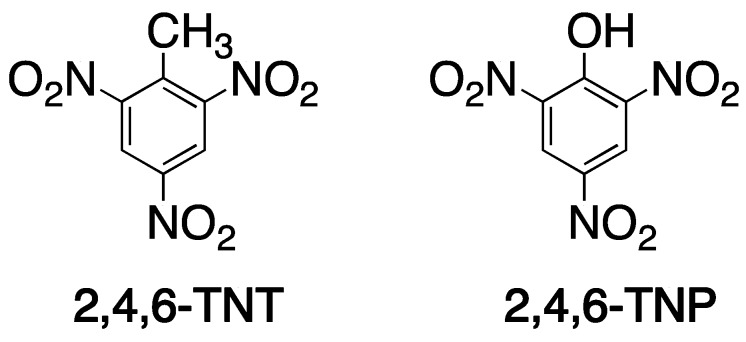
TNT and its less explosive analog TNP.

Once the polymerization process is complete, a subsequent template removal is required to free the imprinted sites [57]. Large templates [12] and the highly cross-linked nature of MIPs [66] together have negative impacts on the release of template molecules [43,67]. An inefficient template removal results in subsequent “template leakage” or “template bleeding” that may contribute to false positive signals in sensing [2] and poor separation in chromatography [68]. Approaches to solve these problems include the use of dummy templates and surface imprinting [12]. To date, the dummy imprinting strategy using a template structural analog (auxiliary template) produces satisfactory outcomes [69,70]. Moreover, the dummy template represents an effective solution in cases when the original template is a dangerous or unstable material, or is very expensive [71,72]. An example of such a case lies in the development of MIPs selective for TNT, which were fabricated using trinitrophenol (TNP) as a dummy template [71]. The resultant MIP demonstrated high sensitivity and selective binding capabilities (Figure 5). Unfortunately, suitable dummy templates are not always available.

Shifting MIP synthesis from bulk to nanoparticles can improve the situation with template leaking as it is easier to extract template from small particles with binding sites located onto or close to the surface [73]. Even so, extraction is a slow procedure, typically performed by dialysis, and in the case of protein imprinting, complete removal of the template is impossible [49].

### 2.3. Heterogeneous and Non-Specific Binding Sites

The problem of high levels of non-specific binding in bulk MIPs is a result of random orientation of the template-monomer complex in the polymer network [74], differences in the nature of the complexation of the template due to the equilibria governing the non-covalent interactions of these complexes and even the presence of template–template interactions [14]. In addition, the relatively high surface areas of imprinted polymers in relation to the number of high affinity sites contribute directly to the non-specific binding observed for the template and interfering molecules [75], a feature which often hinders MIP use in applications such as sensing and separation. To improve the homogeneity of the binding sites, several successful strategies have been suggested such as the semi-covalent approach [76] and stoichiometric non-covalent imprinting [77].

The type of the template has major impact on selecting functional monomers suitable for molecular imprinting. Strong interactions are required to form stable complexes between small organic templates and monomers that can ‘survive’ through the radical polymerization and produce good quality binding sites. However, employing charged monomers to gain a strong monomer-template interaction may lead to very high non-specific binding [68]. These monomers randomly distribute over the surface of the polymer as well as in the imprinted sites and a net negative or positive charge can interact non-specifically with all species carrying the opposite charge. The optimal ratio between monomers and template can be determined empirically [78] or computationally, by using molecular modelling [14,79,80,81]. In the case of protein imprinting, the use of monomers with strong interactions between monomers and template is not recommended. This can be compensated by a multitude of weak bonds formed between protein functional groups and neutral monomers such as acrylamide or weak acids and bases [57]. The nano-format opens another interesting procedure for improving the homogeneity of binding sites. While it is practically impossible to generate perfect binding sites in every single nanoparticle, affinity separation can be used to remove low affinity nanoMIPs from the population of high affinity nanoparticles [82], thus increasing the number of high affinity sites per unit surface area. This approach is similar to the affinity separation of polyclonal antibodies [82]. 

To summarize, bulk imprinting, specifically in relation to imprinting of proteins, has numerous issues that cannot be easily resolved without turning to the nano format, as this format offers solutions to problems associated with imprinting of proteins [83], template leaking [84], and heterogeneity of imprinted sites [85].

## 3. Synthesis of MIP Nanoparticles

**Figure 6:**
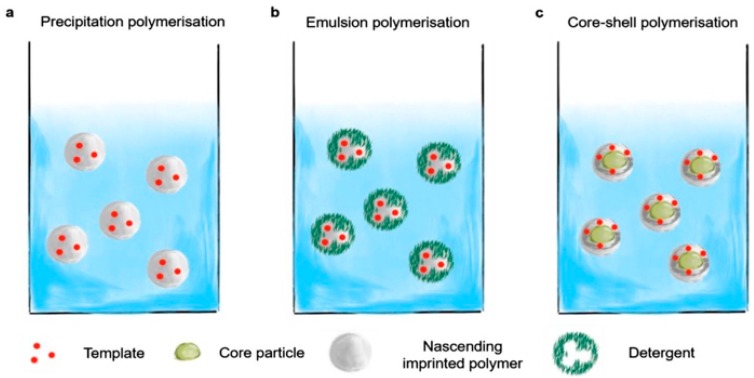
Several protocols used in preparation of nanoMIPs: (**a**) precipitation, (**b**) emulsion, (**c**) core–shell [2,92].

The synthesis of nanoMIPs with precise dimensions and properties is a goal towards which significant steps have been made. While the general theory of controlled radical polymerization is well advanced [86], it is less developed for cross-linked materials such as MIPs. There are very few publications describing modeling of polymerization processes related to MIP synthesis, and studies showing relationships between polymerization parameters and size of cross-linked nanoparticles [87,88]. There are however numerous publications describing empirical syntheses of nanoMIPs with excellent recognition properties [89,90]. The most popular techniques used in nanoMIPs synthesis include precipitation polymerization, emulsion polymerization, and core–shell polymerization with subsequent grafting [91] (Figure 6).

### 3.1. Precipitation Polymerization

Precipitation polymerization is a very promising technique for producing uniform sub-micrometer imprinted particles [12,15,93,94]. It was firstly described by Ye et al. [95] when used for the imprinting of 17ß-estradiol and theophylline. In this method the imprinting process occurs in an excess of solvent. The growing polymer continues to catch oligomers [96] and functional monomers from the solution until they reach critical size leading to precipitation [97,98,99]. The polymer beads are recovered by washing and centrifugation [27]. The advantage of this approach lies in the fact that there is no need for a stabilizer as the MIP particles show no coalescence by virtue of their cross-linking and rigidity [98]. Several factors such as solvent polarity, temperature and stirring speed have a great impact on the MIP particle size [100,101,102]. The main problem associated with this method is the requirement for a large quantity of template dissolved in the excess of solvent [12,27,44]. The high dilution factor may subsequently decrease the interactions between templates and the functional monomers leading to a reduction in the product’s selectivity and sensitivity [91]. Overall this approach is poorly controlled, expensive, and time consuming [12,44].

### 3.2. Emulsion Polymerization

Particles of sub-micrometer scale can also be prepared by emulsion polymerization [2]. Commonly, polymerization is performed in oil-in-water emulsions (O/W) in the presence of surfactant [103,104,105]. This technique can be performed in the form of mini and micro-emulsion polymerization. In the mini emulsion method, a high yield of homogenous [2] MIP-nanoparticles of 30–500 nm diameter can be obtained by stabilizing the monomer droplets (50–1000 nm) in water using co-surfactant (e.g., hexadecane and cetyl alcohol) along with a suitable surfactant, to suppress the diffusion processes in the aqueous phase [17]. In order to disperse the two phases, high-shear homogenization is required either by vigorous stirring and/or sonication [103]. However, the presence of water and surfactants can have adverse effects on the formation of stable monomer-template complexes. Micro-emulsion polymerization can yield particles of 5–50 nm diameter [17]. It is performed in a thermodynamically stable emulsion formed in the presence of co-surfactant by a high shear homogenization step [106]. This system requires higher surfactant concentration and lower monomer concentration than that used in the mini-emulsion method [17]. Generally speaking, all emulsion approaches suffer from the use of chemicals, in particular surfactants, that interfere with molecular recognition, contaminate the polymeric product and require complicated and time-consuming purification steps.

### 3.3. Core–Shell Grafting and Polymerization

Principally, there are two stages required to obtain core-shell MIP nanoparticles. The first one is the formation of solid nanocore (seed particle) while the second stage is the grafting of the imprinted shell [107]. The solid core can be formed from diverse materials containing additional functionalities suitable for anchoring e.g., catalytic groups capable of initiating polymer grafting [108,109]. This approach allows formation of recognition sites at the surface of MIP beads [107,110] improving analyte transfer [111,112]. Grafting of a thin imprinted layer on prefabricated nanoparticles [113] is preferred over emulsion polymerization in the presence of seed particles [17] since it allows better control of the thickness of imprinted film [114]. In addition, grafting using iniferter chemistry allows post functionalisation of synthesized nanoparticles with fluorescent, PEG, or anchoring groups [115,116].

### 3.4. Solid Phase Imprinting

**Figure 7:**
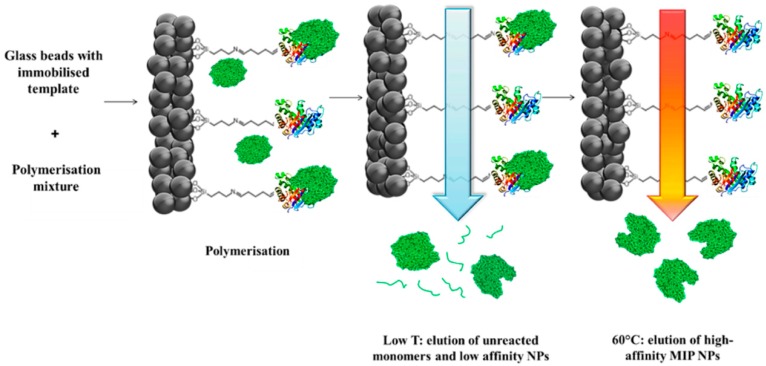
Schematic representation of solid phase synthesis of nanoMIPs.

This technique is considered as one of the most advanced approaches for the fabrication of nanoMIPs. According to Canfarotta et al. [84], the solid phase method consists of three main steps: preparation of the glass beads by activation and silanization, immobilization of the template on the silanized glass beads, and finally the polymerization and purification process (Figure 7).

The advantages of solid-phase imprinting include: the possibility for re-using templates attached to the solid phase, orientational control of template-polymer interaction contributes to a more homogeneous distribution of recognition sites, the ‘inbuilt’ affinity purification yields high affinity nanoMIPs, products are virtually free from templates which eliminates bleeding issues.

## 4. Applications of Nano MIPs

The main advantages of nanoMIPs as compared with antibodies, aptamers and other biomimetics include:

- High stability against non-physiological conditions such as high temperature, extreme pH, and pressure [6,22].
- Size of these nanoparticles is comparable to those of proteins and they have high apparent binding constants.
- NanoMIPs can be stored at room temperature for very long time [117].
- Synthesis of nanoMIPs requires weeks instead of months as in the case of antibodies [92].
- NanoMIPs can be easily functionalized with fluorescent, catalytic, or magnetic labels [17,118].
- NanoMIPs can pass the cell membrane barrier and be delivered to cell targets orally [30].
- While it has not yet been demonstrated in practice, the production of nanoMIPs has the potential to be more economical than that of antibodies [119].

### 4.1. NanoMIPs in Separation

The separation field is continuously expanding driven by the needs of the pharmaceutical and chemical industries, and by the demands for water purification and waste remediation. By virtue of their high affinity and selectivity, MIPs have been employed as stationary phases in high performance liquid chromatography [120], capillary chromatography [121,122], and solid phase extraction [123]. The nano format however is not ideally suited for large-scale separation. The main barrier for this is the relatively low binding capacity of nanoMIPs, especially monoclonal nanoparticles, and their high price. In addition, nanoparticles have to be covalently immobilized onto solid support for chromatographic application, and this procedure can potentially affect their recognition properties. To solve this problem, nanoMIPs were fabricated with a magnetic core which facilitated their handling during extraction of tetracycline antibiotics [124]. In some other rare examples of separation, applications nanoMIPs have been successfully employed in capillary electrophoresis [125,126].

### 4.2. NanoMIPs in Catalysis

MIPs with catalytic properties can be considered as suitable alternatives for natural enzymes [127]. MIP-based catalysis continues to evolve as one of the most interesting challenges [128]. To date, the main success with creation of catalytic sites in nanoMIPs is related to imprinting of transition state analogs (TSA) of catalytic reactions [129,130]. In these examples, nanoMIPs demonstrated very impressive catalytic constants and turnover. However, despite demonstrating proof-of-concept, no examples have yet been reported that address problems related to real (industrial) applications and for the present, this application remains a scientific curiosity similar to catalytic antibodies [131].

### 4.3. NanoMIPs in Assays and Sensors

Microtiter plate-based assays are very important for clinical and environmental analysis [132,133]. Enzyme-linked immunosorbent assay (ELISA) is among the most common methods of quantification of analytes in various complex samples. Its principle is based on exploring recognition properties of natural antibodies and enzymatic amplification of the signal [134]. In total ~12,000 assay protocols are available in the PubChem BioAssays database. However, traditional assays generally include between 7 and 10 time consuming liquid-handling steps and take three to five hours to produce a result [135]. Other drawbacks of ELISA include relatively limited detection range due to the narrow sensitivity of monoclonal antibodies, their low stability and high cost of production. The intrinsic low stability of antibodies has a big negative impact on shelf life of the manufactured bioassays and requires a constant cold chain supply. It also limits the operation conditions to a very narrow physiological range. Production of antibodies is a long process, which takes 3–6 months. There is a specific requirement for ‘good’ antigens to be at least 6,000 Da in order to generate good antibodies. The antibody production for small molecules includes synthesis of a conjugate consisting of a carrier molecule, usually a protein, and a molecule of interest [79]. Sometimes antibodies are not available due to the high toxicity of the target molecule, as in the case of the mycotoxin patulin [79]. For these reasons, there are continuous R&D efforts to develop robust, more rapid and more sensitive assay systems.

Several attempts over the years were made to use MIPs in the assays [136,137]. The obvious advantage of MIPs is their ability to recognize small molecules, including toxins [138,139]. Historically, MIPs were prepared using the bulk polymerization approach and were ground to micrometer size for use in the assays [140]. A problem commonly associated with this approach is the difficulty with reproducible immobilization of MIPs in microplate wells. Several attempts were made to avoid the problem with poor reproducibility of immobilization protocols in the development of MIP-based homologous assays using competition with a fluorescently labeled template [141,142,143,144]. Since MIPs used in these examples were in the form of microparticles, they did not form stable suspensions in solution, which made their practical application unreliable. Only with the development of effective synthesis of MIP nanoparticles using a solid phase approach [145] did it become possible to prepare MIP nanoparticles or ‘plastic’ antibodies for practically any analyte of interest and integrate them with microtiter plates by standard protocols used in antibody-based ELISA. In a first such example, MIP nanoparticles for vancomycin were physically immobilized on the surface of a microtiter plate and all other steps were conducted as in the classical ELISA [146]. It was demonstrated that the developed assay was able to detect the antibiotic vancomycin in buffer and in blood plasma within the range of 0.001−70 nM with a detection limit of 2.5 pM. Other examples of pseudo-ELISA assays based on MIP nanoparticles describe assays for cocaine [147] and gentamicin [148] with very similar sensitivity for relevant targets. Attractive features of the new assays included long shelf life, lower manufacturing costs, and a short production time. 

**Figure 8:**
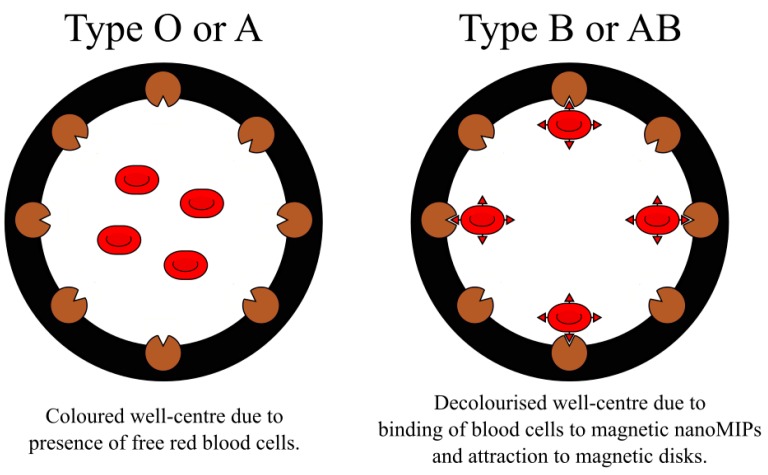
Responses of blood type B or AB to the presence of blood type B-specific MIP nanoparticles. The black circles are magnetic disks on the inside walls of the microtiter plate wells.

Another important aspect in the development of abiotic assays featuring MIP nanoparticles was the introduction of magnetic inserts in the wells of the microtiter plate [149]. The innovation involved the use of disk-shaped inserts made of magnetic material, which quickly and effectively captured any paramagnetic nanoparticles with immobilized target analytes. The internal opening of the disks allowed measurements of the reactions using standard microtiter plate readers. One of the examples of such application demonstrated that magnetic nanoMIPs imprinted with blood type B trisaccharide (Gal-⍺-1,3(Fuc-⍺-1,2)Gal) could be used in blood typing assays instead of natural antibodies [150]. The new format of the test system was based on magnetically-induced decolorization, caused by removal of erythrocytes from the solution by magnetic nanoMIPs (Figure 8).

**Figure 9:**
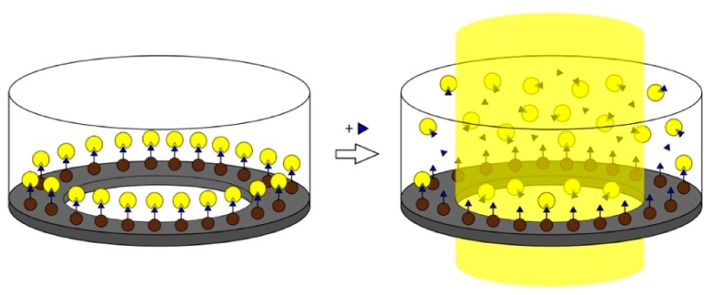
A schematic representation of the MINA process: the microtiter plate well is coated with fluorescent nanoMIPs (**yellow**) linked to biotin (**blue**). The biotin is fixed on magnetic nanoparticles (**brown**). By addition of free target species to the tested well, a displacement of the nanoMIPs occurs with subsequent diffusion to the center enhancing the fluorescent signal. Reproduced from [152] with permission.

A similar principle is used in the magnetic imprinted nanoparticle-based assay (MINA) for detection of methyl parathion [151], biotin [152], and proteins [153]. This assay was based on competition of fluorescent nanoMIPs for binding to free analyte and analyte immobilized on magnetic inserts (Figure 9). The presence of free analyte in solution led to increase in fluorescence, with a biotin limit of detection of 7 nM [152].

**Figure 10:**
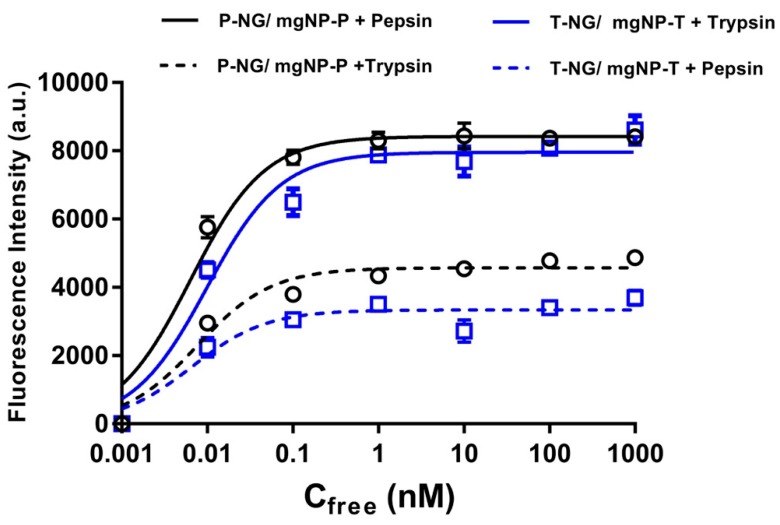
Fluorescence intensity of nanoMIPs displaced from the corresponding magNPs upon addition of incremental amounts of protein and incubation for 2 h. The excitation/emission filters used were 485 and 520 nm, adapted from data presented by Mahajan et al. [153].

Recently, an integrating approach for the synthesis and direct assay for protein-imprinted nanoMIPs has been reported using magnetic nanoparticles [153]. The enzymes trypsin and pepsin were immobilized on solid support, i.e., functionalized magnetic nanoparticles (magNPs). Subsequently, lightly crosslinked fluorescently doped polyacrylamide nanoMIPs were produced in the presence of the magNPs. The nanoMIPs were then used in a magnetic competitive fluorescence assay employing identical protein-conjugated magNPs as ligands to reversibly immobilize the corresponding nanoMIPs. Both nanoMIPs exhibited *K*_d_ < 10 pM for their respective target protein and low cross-reactivity was observed for reference proteins (Figure 10).

The advantages of this assay format, as compared with ELISA, include the use of highly stable reagents and simple protocols with minimal operational steps that do not require highly experienced operators [152]. MINA assays are characteristically very effective, and can in principle be developed for any template of interest, moreover they are capable of working in any complex media, such as milk or blood plasma. They have a long shelf-life and do not depend on availability of a cold chain supply.

Sensors are probably the most advanced (and attractive) niche application for imprinted polymers. More papers were published on MIP sensors than any other types of sensors based on synthetic receptors (Web of Science, accessed 10 December 2019). Representative examples include MIP-based QCM sensors for human rhinovirus immunoglobulins [154], tobacco mosaic virus [155], and *Salmonella paratyphi* [34]. MIP films were deposited on the sensor surface via surface grafting [156,157] or electropolymerization [158,159]. MIPs were incorporated into QCM sensors [160], optical sensors [161,162], and electrochemical sensors [163]. The advantages offered by nanoMIPs over other imprinting formats make them interesting targets for use in other sensing configurations not least surface-based techniques—e.g., QCM, SPR—where high densities of recognition sites close to the transducer is most desirable.

### 4.4. NanoMIPs in Life Science and In Vivo Applications

In vivo applications demand MIPs in the form of nanoparticles. For such applications it is also necessary to demonstrate that nanoMIPs are not toxic, and do not interfere with cell ‘machinery’. There is a limited body of evidence that suggests that nanoMIPs are not toxic in cell culture [164]. Interestingly, nanoMIPs were able to cross cell membranes, which indicates an opportunity for pursuing intracellular targets for therapeutic applications. NanoMIPs selective for melittin showed no toxicity in fibrosarcoma cell culture over the tested concentration range (3−3000 µg·mL^−1^). When tested with mice, the same nanoMIPs showed no toxicity two weeks after injection in histopathological examination of liver, lung, and kidney [138,139]. There are three most obvious areas for possible application of nanoMIPs in vivo: toxin scavenging, imaging, and drug delivery. In a first study of such nature, Hoshino and colleagues have tailored imprinted nanoparticles against melittin, the active component of the bee venom. When the melittin-imprinted polymer was injected into living mice, it was acting as an effective antidote via capturing and clearing melittin from blood circulation. Significantly, both toxic symptoms and mortality were greatly diminished [138,139].

An exciting area for nanoMIP application is in targeted drug delivery. Recently, a series of reports have been published describing the use of nanoMIPs as drug carriers for insulin, (*R*)-thalidomide, carbazole derivatives, quercetin, and paclitaxel anti-cancer drugs [30,165,166,167,168]. The drug release was achieved using either photo-, thermo-, or pH-responsive stimuli [169,170,171,172,173]. For imaging applications nanoMIPs can be easily functionalized either with quantum dots or fluorescent dyes [139,174,175]. Kunath, et al. [176] targeted hyaluronan molecules on cell surfaces of fixated and living tissues. In this work, they used fluorescence for detecting the presence of dye-labelled nanoMIPs in the epidermal basal layer and papillary dermis. A very significant step towards targeted drug delivery can be found in the recent report by the Liu group [177] describing HER2 N-glycan nanoMIPs, which were shown in in vitro studies to inhibit HER2+ cell proliferation. Importantly, in vivo studies demonstrated the attenuation of HER2+ breast cancer tumor growth by approximately 50% relative to control groups, highlighting the potential of nanoMIPs in therapeutic applications.

## 5. Conclusions and Future Outlook

It has been shown that nanoMIPs possess superior properties to bulk polymers in many respects, including affinity, specificity, and their ease of integration into assays and sensor formats. The soluble nature of these materials opens for their possible use in in vivo applications such as imaging and drug delivery, areas which are being pursued by us and others. The remaining challenges associated with this technology include: (i) lack of evidence of commercial success for MIP-based assays and sensors; (ii) requirement for comprehensive analysis of nanoMIP toxicity, biodistribution and clearance; and (iii) demonstration of comparable performance of MIP nanoparticles to antibodies in key therapeutic applications, such as immunotherapy. We believe that these developments shall be in part driven through fundamental studies of these novel materials. Resolution of these challenges shall undoubtedly open for a broader interest for using of these materials in a range of applications where antibodies or biomolecular receptors are currently used.
